# Multimodal Machine Learning for Predicting Post-Surgery Quality of Life in Colorectal Cancer Patients

**DOI:** 10.3390/jimaging10120297

**Published:** 2024-11-21

**Authors:** Maryem Rhanoui, Mounia Mikram, Kamelia Amazian, Abderrahim Ait-Abderrahim, Siham Yousfi, Imane Toughrai

**Affiliations:** 1 Laboratory Health Systemic Process (P2S), UR4129, University Claude Bernard Lyon 1, University of Lyon, 69008 Lyon, France; 2 Meridian Team, LyRICA Laboratory, School of Information Sciences, Rabat 10100, Morocco; 3 Higher Institute of Nursing Professions and Health Technology, Fez 30050, Morocco; 4 Human Pathology, Biomedicine and Environment Laboratory, Faculty of Medicine and Pharmacy, Sidi Mohamed Ben Abdellah University, Fez 30000, Morocco; 5 General Surgery Department, Hassan II University Hospital, Fez 30050, Morocco

**Keywords:** multimodal learning, machine learning, quality of life (QoL), colorectal cancer (CRC), healthcare analytics

## Abstract

Colorectal cancer is a major public health issue, causing significant morbidity and mortality worldwide. Treatment for colorectal cancer often has a significant impact on patients’ quality of life, which can vary over time and across individuals. The application of artificial intelligence and machine learning techniques has great potential for optimizing patient outcomes by providing valuable insights. In this paper, we propose a multimodal machine learning framework for the prediction of quality of life indicators in colorectal cancer patients at various temporal stages, leveraging both clinical data and computed tomography scan images. Additionally, we identify key predictive factors for each quality of life indicator, thereby enabling clinicians to make more informed treatment decisions and ultimately enhance patient outcomes. Our approach integrates data from multiple sources, enhancing the performance of our predictive models. The analysis demonstrates a notable improvement in accuracy for some indicators, with results for the Wexner score increasing from 24% to 48% and for the Anorectal Ultrasound score from 88% to 96% after integrating data from different modalities. These results highlight the potential of multimodal learning to provide valuable insights and improve patient care in real-world applications.

## 1. Introduction

Colorectal Cancer (CRC) is the third most common cancer worldwide. In 2020, an estimated 19.3 million new cancer cases were reported, with breast cancer leading at 11.7%, lung cancer at 11.4%, and CRC at 10.0% [1]. CRC can occur in the colon or rectum and typically begins as a benign polyp, which can develop into a malignant tumor over time. Traditional treatments for CRC, including surgery, radiation, and chemotherapy, have significantly improved survival rates. However, these treatments often result in surgical trauma or damage to the pelvic nerve plexus, leading to dysfunctions that can greatly impact the quality of life (QoL) of patients [2].

The healthcare industry is facing an increasing demand for services, resulting in a massive amount of data generated by hospitals daily. These data, often overlooked due to the busy nature of healthcare professionals, hold immense potential for insights and optimization when analyzed using artificial intelligence (AI) [3]. AI’s application in healthcare has the potential to revolutionize the industry by enabling disease detection without relying on expert input and providing predictive insights into patient conditions. This could significantly enhance the healthcare system and inform decision-making within the medical domain.

AI is transforming the healthcare industry by improving patient outcomes and operational efficiency. AI algorithms can analyze vast amounts of medical data and images to assist in disease diagnosis [4] and treatment planning, predict future health conditions and disease risk, assist in the discovery of new drugs, improve medical imaging accuracy [5], manage electronic health records, and provide real-time decision support to healthcare providers.

Among the many AI-driven approaches, deep learning techniques like convolutional neural networks (CNNs) have shown particular promise in medical image analysis. CNN-based models, such as U-Net [6], have become foundational for tasks requiring pixel-wise predictions, such as image segmentation and density map regression. Density map regression, which involves predicting continuous values over an image, has been effectively used in medical imaging for tasks like organ [7,8] and tumor [9,10] volume estimation, where precise localization and counting are critical.

Recent years have witnessed a surge in research articles focusing on early detection [11] and prognostic outcomes of colorectal cancer [12], driven by advancements in artificial intelligence and machine learning. A particular area of interest is the impact of curative resection operations on a patient’s QoL, due to the predictive capabilities of machine learning algorithms.

Multimodal machine learning [13], which integrates data from multiple sources, can be extremely valuable in critical fields such as finance [14], security [15], and ultimately healthcare [16]. In healthcare, this approach enhances our understanding of a patient’s health by incorporating diverse information such as clinical, genetic, and imaging data. This leads to more accurate diagnoses and personalized treatment plans. In addition, multimodal machine learning can facilitate the analysis of large and complex datasets, increasingly common in healthcare due to the widespread adoption of electronic health records. It can also enable the development of automated systems for detecting patterns and identifying important trends, saving time and improving efficiency. By significantly improving the quality of healthcare, multimodal machine learning makes advanced care more accessible to a broader range of patients.

This paper aims to predict various factors influencing CRC patients’ QoL by employing a multimodal machine learning framework that integrates diverse data sources. This study focuses on indicators such as the Global Quality of Life Index (GQOLI2), Wexner Score, Low Anterior Resection Syndrome (LARS) Score, and Anorectal Ultrasound (AUR) Score [17]. These indicators collectively provide a detailed picture of the patient’s post-treatment life and are crucial for guiding clinical decisions that could significantly enhance patient outcomes and overall well-being.

The remainder of this paper is organized as follows: Section 2 explores the context and motivations behind this study. Section 3 reviews related works. Our proposed approach is outlined in Section 4. Finally, Section 5 discusses the future works and conclusions.

## 2. Motivation and Quality of Life Indicators in Colorectal Cancer

Colorectal cancer management has evolved significantly in recent years, with a growing recognition that patient care extends beyond survival rates alone. Quality of life has become a crucial focus in cancer care [18], reflecting a shift toward more holistic and patient-centered approaches. This emphasis on quality of life is motivated by the understanding that a patient’s overall well-being profoundly influences treatment outcomes, recovery processes, and long-term satisfaction with care [19].

Despite advancements in CRC treatment modalities, patients frequently encounter substantial physical and psychological challenges post-treatment. These challenges underscore the need for comprehensive, proactive approaches to assessing and managing quality of life in CRC care. Key motivations for this study include the following:

- Imitations of Current QoL Assessments: Traditional methods, typically involving post-surgical questionnaires, often provide delayed insights that fail to inform real-time clinical decision-making, limiting the ability to preemptively address potential QoL issues.
- Gap in Predictive Care Models: There is a lack of robust predictive models to anticipate QoL outcomes before surgical interventions, which could significantly improve treatment planning and patient preparation.
- Untapped Potential of Healthcare Data: The vast amount of healthcare data in CRC management remains largely underutilized for predictive and personalized care approaches.
- Need for Personalized Treatment Strategies: Each CRC patient’s journey is unique, yet current treatment protocols lack the granularity to address individual QoL concerns effectively.
- Improving Informed Consent and Patient Expectations: More accurate predictions of post-treatment QoL could significantly enhance the informed consent process, helping to set realistic expectations and improve patient satisfaction.
- Enhancing Surgical Decision-Making: Predictive tools for QoL outcomes would enable surgeons and oncologists to make more informed decisions about surgical approaches and post-operative care strategies.

In addressing these motivations, this study utilizes the following key quality of life indicators to quantify and monitor the impact of colorectal cancer treatments:

- GQOLI2: A broad measure of a patient’s subjective well-being across multiple life domains, offering insights into psychosocial adaptation post-treatment.
- Wexner Score: Assesses the severity of fecal incontinence, a common issue following colorectal cancer treatment, crucial for understanding its social and psychological impact.
- LARS Score: Quantifies bowel dysfunction symptoms experienced by patients after anterior resection, providing insights into functional recovery and needed interventions.
- AUR Score: Evaluates structural outcomes post-treatment using anorectal ultrasonography, linking physical changes to their impact on quality of life.

By introducing these indicators in a multimodal machine learning framework, this study seeks to identify key predictive factors for each quality of life indicator, thereby enabling clinicians to make more informed treatment decisions and develop personalized care strategies.

## 3. Related Works

In recent years, the medical field has greatly benefited from advancements in AI, optimizing both time and cost in various domains, including patient diagnosis and expert decision-making support. This progress has significantly impacted medicine, with one notable area being the prediction of QoL after CRC resection. This section explores and synthesizes the current state of the art in this field (Table 1).

Breukink et al. [20] conducted a prospective study to evaluate the QoL in rectal cancer patients who received laparoscopic total mesorectal excision surgery over a one-year period. Using the European Organization for Research and Treatment of Cancer Quality of Life Questionnaire (EORTC QLQ), data were collected at 3, 6, and 12 months post-surgery. The assessment included five functional scales (physical, role, cognitive, emotional, and social) and three symptom scales (fatigue, pain, and nausea/vomiting), along with several single-item symptoms common among colorectal cancer patients, such as dyspnea, loss of appetite, insomnia, constipation, and diarrhea. This study found an overall improvement in QoL one year after surgery, although there was a notable decline in specific areas, particularly in physical and sexual functioning.

Gray et al. [21] investigated factors associated with CRC that could potentially explain variations in QoL after diagnosis. Utilizing the EORTC QLQ questionnaire and multiple linear regression models, this study aimed to identify key predictors of QoL in CRC patients. The findings indicated that gender, an unmodifiable factor, significantly affects QoL, with females generally experiencing poorer outcomes than males. In contrast, modifiable factors such as social functioning, fatigue, dyspnea, and depression were strong predictors of diminished QoL. The multiple linear regression model developed for predicting global QoL demonstrated robust performance, achieving an R-squared score of 0.574 using these identified factors.

Xu et al. [22] highlights the significance of CRC as the third leading cause of tumor mortality with a 5-year survival rate of approximately 40%, mostly due to postoperative recurrence and metastasis. Surgical treatment with adjuvant therapy is the main clinical approach for CRC. However, the high recurrence rate of 80% three years after surgery negatively impacts patients’ lives. Thus, this study aims to investigate the feasibility of machine learning in predicting CRC recurrence after surgery. Four machine learning algorithms, namely Logistic Regression, Decision Tree, Gradient Boosting, and LightGBM, were implemented. The results reveal that the Gradient Boosting and LightGBM models outperform the other two algorithms. This study also identifies age and chemotherapy as the most influential risk factors for tumor recurrence.

Valeikaite et al. [23] analyzed predictors of long-term QoL in 88 CRC patients who received curative operations in Lithuania. QoL was assessed pre-surgery and at 1, 24, and 72 months post-surgery using standardized questionnaires. Statistical analyses included non-parametric tests and multivariate logistic regression models. This study found a 67% 6-year survival rate, with positive long-term outcomes in CRC treatment and stable QoL observed two years post-surgery. Emotional and social deficits emerged as the main issues for patients, while age and radiotherapy were associated with decreased long-term QoL. This study suggests that interventions targeting these factors could help improve long-term QoL outcomes.

Lu et al. [24] conducted a review aimed at evaluating studies that identify patients at risk of short-term mortality, to improve QoL and reduce cancer recurrence. The researchers analyzed 15 articles from various databases, finding that 12 of these studies had a high risk of bias due to small sample sizes or reliance on single metrics. The reviewed articles covered multiple types of cancer and varied widely in terms of predictors and sample sizes used. Common data pre-processing techniques included discretization, normalization, and handling of missing data, while feature selection methods included recursive feature selection, parameter-increasing selection, and forward-step selection. This study highlights limitations in the existing research, particularly regarding sample size and methodological consistency, and provides insights into the techniques employed for data analysis.

Sim et al. [25] evaluated the role of HRQoL in predicting 5-year survival among lung cancer patients. The study compared two feature sets: one containing only clinical and sociodemographic variables, and the other incorporating additional QoL factors, as many lung cancer survivors reported health challenges. The results indicated that the second feature set, which included HRQoL factors, demonstrated higher predictive performance, suggesting that QoL plays a crucial role in determining patient survival.

Savic et al. [26] explored the variability in QoL among cancer patients, focusing on predicting QoL specifically for breast and prostate cancer patients. The study employed two datasets and compared two approaches: centralized learning, wherein data are combined before model training, and federated learning, which allows for machine learning models to be trained collaboratively without direct data sharing.

Karil et al. [30] developed a machine learning model to predict declines in HRQoL between 12 and 60 months post-tumor resection. Using the Quality of Life Questionnaire-Core 30 (QLQ-C30) to assess HRQoL, the model’s target class was binary, indicating the presence or absence of HRQoL decline. Selected features included patient demographics (age, gender, relationship status), tumor characteristics (lateralization, histological diagnosis, extent of resection), and prior treatments such as radiotherapy and chemotherapy. Although the model achieved promising results, the authors highlighted limitations due to the small dataset size and recommended further data collection to improve the model’s generalizability.

The studies reviewed in this section highlight AI’s potential to improve QoL predictions following CRC resection. While statistical analysis and machine learning/deep learning models have shown promise in forecasting QoL and tumor recurrence, there remains significant room for improvement. More comprehensive studies utilizing advanced models could yield deeper insights into the factors influencing patient QoL after CRC surgery.

Most techniques in the literature rely on a single modality, such as questionnaires or imaging data, which limits predictive accuracy by depending on only one source of information.

In contrast to previous studies that focus on either clinical or imaging data in isolation, our research introduces a multimodal approach that integrates both types of data. This combined approach seeks to capture synergistic insights often missed in single-modality studies, potentially enabling more accurate predictions of post-treatment QoL outcomes for CRC patients.

## 4. Methodology

**Figure 1:**
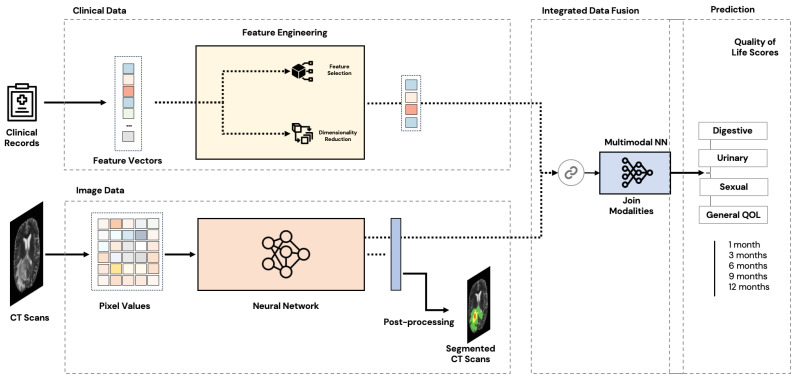
General multimodal architecture.

Figure 1 illustrates the proposed general multimodal architecture.

### 4.1. Data Collection and Pre-Processing

The data used in this study are multimodal, including clinical data with several features and segmented CT Scans for the corresponding patients. The dataset contains information on 100 CRC patients.

#### 4.1.1. Data Collection

Data were sourced from CHU Hassan II University Hospital (Fès), Morocco, under ethical approval and with full anonymization to ensure patient privacy. The dataset includes clinical data and CT scan images from 100 CRC patients, recorded at multiple time points: pre-surgery, immediately post-surgery, and at 3, 6, and 12 months post-surgery.

- **Clinical Data**: Collected from electronic health records, the clinical dataset contains 49 features, including patient demographic information (age, gender), medical history (e.g., hypertension, diabetes, smoking status), treatment details (surgery type, chemotherapy, radiotherapy), and post-surgical outcomes (complications, recurrence rates). The longitudinal follow-up allowed us to track changes in the quality of life indicators such as the Wexner Score, LARS Score, and AUR Score over time.
- **Imaging Data**: The CT scan images, collected at each follow-up point, provide detailed views of the patients’ anatomy. These images focus on tumor morphology and location. Tumor segmentation masks were manually annotated by medical professionals, highlighting the regions of interest for our analysis.

Our study utilizes a longitudinal dataset with a one-year follow-up period for each patient, which significantly impacts the sample size. The extended follow-up is crucial for accurately assessing post-surgical quality of life outcomes in CRC patients, as many complications and changes in QoL indicators manifest over time.

The sample of 100 patients strikes a balance between the depth of data collected and the breadth of the cohort. Each patient in the study participated in multiple assessments over the course of a year, including:

- Pre-surgical baseline measurements;
- Immediate post-surgical evaluations;
- Regular follow-up assessments at 3, 6, and 12 months post-surgery.

This intensive data collection process yielded a rich, multi-timepoint dataset for each patient, allowing us to capture the dynamic nature of QoL changes over time. The comprehensive nature of these data, while limiting the total number of patients we could include within the study timeframe, provides valuable insights into the trajectory of QoL indicators. This approach enhances the statistical power of our analyses, partially mitigating the limitations of a smaller sample size.

The study was conducted in accordance with the Declaration of Helsinki and approved by the Ethics Committee of the Faculty of Medicine and Pharmacy of Fez (protocol code N 27/20, September 2020). All patients provided written informed consent. To protect patient privacy, all data were de-identified before analysis.

#### 4.1.2. Clinical Data Acquisition

Depending on the analysis focus, this dataset comprises real-world data from 100 CRC patients, including up to 49 features and potentially up to 106 target variables. Table 2 offer a detailed description of each variable.

#### 4.1.3. Pre-Processing

Data cleaning involves removing noise that could cause model failure, such as missing values and outliers. Important fields were handled with care by experts. The process included the following:

1. Handling missing data by dropping columns with high missing values and imputing others using K-Nearest Neighbor algorithm [32].
2. Handling outliers by correcting erroneous records with the help of experts.
3. Discretizing continuous variables based on established thresholds to evaluate QoL.
4. Balancing data by using Synthetic Minority Oversampling Technique (SMOTE) to generate synthetic samples and Random Oversampling for targets with insufficient samples.
5. Scaling data to unify ranges and prevent confusion for the model.
6. Encoding labels to handle numeric data and start from 0 rather than 1 for some algorithms.

#### 4.1.4. Feature Engineering

To address the high number of features in the dataset, we used two approaches. Firstly, we employed various feature selection algorithms, including backward and forward selection, as well as domain-specific algorithms optimized for medical classification tasks. Secondly, we used principal component analysis to combine features into components while maintaining the maximum amount of explained variance. The feature selection algorithms work by iteratively adding or removing features to the model based on their significance, while the principal component analysis reduces the dimensions of the features by combining them into components. The feature selection algorithms include forward selection, which adds features iteratively until no further improvement is observed or a maximum number of features is reached, and backward selection, which eliminates features iteratively until the maximum number of features is reached. Additionally, we used various optimization algorithms [33,34,35,36] to optimize the feature selection process.

#### 4.1.5. Imaging Data Acquisition

The imaging data in the dataset consist of CT Scans of the patients, which were collected using various imaging techniques. The CT scans provide detailed information on the structure and morphology of the tumor, as well as its location and extent.

While various imaging modalities have been used in CRC research, including MRI and PET scans, we focus on CT images for several reasons. CT scans offer high spatial resolution, are widely available, and provide detailed information about both soft tissues and bony structures. Furthermore, CT is the standard imaging modality for staging and follow-up in CRC patients.

#### 4.1.6. Pre-Processing

First, we resized the noisy images and cropped uninformative black areas using contour detection. Then, we reduced noise using the CLAHE equalization technique and generated segmentation masks for red contours by filtering non-red colors, as defined by experts.

### 4.2. Clinical Data Analysis

#### 4.2.1. Digestive Troubles Analysis

Table 3 shows the performance results of various machine learning models on predicting digestive troubles, as measured by the F1-score and accuracy metrics. The results are presented for different time periods, ranging from 1 month to 9 months after surgery, and for two different targets: LARS score and Wexner score. The models used include Random Forest, LightGBM, Logistic Regression, Decision Tree, K-Nearest Neighbors, and Gradient Boosting.

The AUR score depends on the nature of surgery on early months and somewhat on the tumor field on later months. On later months, the LARS score is heavily affected by the field that was occupied by the tumor. The Wexner score is also severely affected by the field of the tumor on later months.

#### 4.2.2. Urinary Troubles Analysis

Table 4 presents the results of different machine learning models trained to predict urinary troubles in patients at different time points. The target variable includes UI score and AUR score. The models used include XGB (extreme gradient boosting), random forest, decision tree, and gradient boosting.

Model performance, based on the F1-score, ranged from 0.21 to 0.91, with accuracy spanning 70% to 97%. Selected features varied across models and included factors such as gender, surgery type, surgical approach, distance, rectal/colonic location, complete clinical response (CCR), syndrome R, endoscopy position (endo), position (TDM/TAP), protocol, anastomosis type, adjuvant chemotherapy (CMTadjuvante), extradigestive tumor presence, and colotomy IGD.

#### 4.2.3. Sexual Troubles Analysis

This section presents the results of various models applied to different sexual troubles (dyspareunia and erection troubles) with different time intervals (3 months, 6 months, 9 months, and 12 months) and their corresponding features selected by the models.

For dyspareunia, as seen in Table 5, the results indicate that the models perform differently based on the time interval. The Bagging model achieved an F1-1 score of 0.18 and 63% accuracy for the 3 months interval with only two selected features (Constipation and Fistula). In contrast, the XGB model achieved an F1-1 score of 0.88 and 97% accuracy for the 12 months interval with only two selected features (Age and Gender). This suggests that the selected features and models significantly affect the performance of the prediction model.

For erection troubles, as seen in Table 6, the models performed well in predicting the troubles with high accuracy and F1-1 scores. The K-Nearest Neighbor model achieved the highest F1-1 score of 0.91 and 97% accuracy for the 3 months interval with six selected features (Gender, Diabetes, Tobacco, syd r, Constipation, and envah). For the 6 months interval, the Random Forest model achieved the highest F1-1 score of 0.93 and 97% accuracy with nine selected features (Cardiopathy, Occlusion, Distance, TR:circumference, envah, resecability, CCR, reservoir, and CMTadjuvante). The Decision Tree models performed well for the 9 and 12 months intervals with F1-1 scores of 0.82 and 0.88, respectively, with only a few selected features.

#### 4.2.4. General Quality of Life Analysis

Table 7 shows the different models that were trained to predict the General QoL at different time intervals (1, 3, 6, 9, and 12 months) using different sets of features. The models used for the prediction are AdaBoost [37], XGB [38], and Gradient Boosting [39].

When we examine the selected features for different time intervals, we notice that specific features play important roles in predicting quality of life at different times. For example, when forecasting QoL at 1 month, features like Age, Gender, Cardiopathy, and Operated are significant. In contrast, when predicting QoL at 6 months, features like Extradig Tumor, pos(TDM/TAP), Anastomosis type, ileotsomie, and CMTadjuvante become important.

During the early months, the age and gender of the individual emerge as key factors in determining the level of GQoL. In contrast, during later months, the impact is influenced by factors related to the tumor field and the treatments administered to the patient.

### 4.3. Imaging Data Segmentation

Medical image segmentation is vital in healthcare by enabling precise delineation and analysis of anatomical structures and pathological regions within medical images.

To extract relevant features from CT images for QoL prediction, we employed a two-step process. First, we used a pre-trained segmentation model to isolate the regions of interest (ROI) in the CT scans. This step helps focus our analysis on the most relevant anatomical structures. Second, we extract high-level features from these ROIs. These features were then combined with clinical data in our multimodal framework to predict QoL indicators.

We apply and explore the effectiveness of UNET models and their variants in accurately delineating anatomical structures and pathological regions within medical images.

#### 4.3.1. Segmentation Models

**Figure 2:**
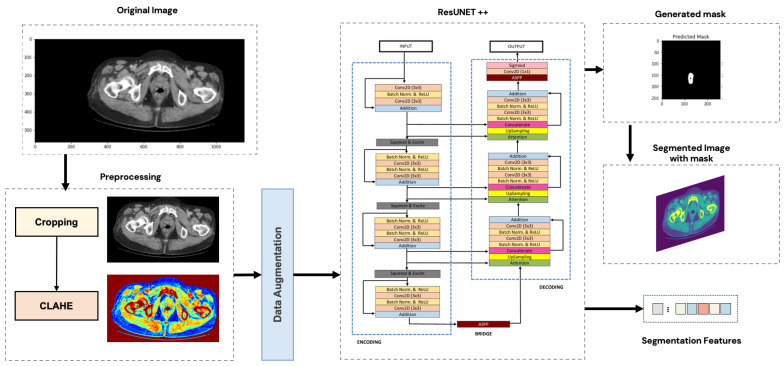
General segmentation approach.

In this section, a succinct overview will be provided on the architecture of the segmentation models. The general segmentation approach is illustrated in Figure 2.

##### U-NET [6]

The U-NET architecture has a unique U-shaped design consisting of two main paths: a contracting or encoding path and an expanding or decoding path. The encoder is a standard convolutional neural network with repeated convolutions and ReLU activations that lead to a feature map. The encoder’s primary goal is to reduce spatial dimensions and increase channels in each layer.

**Figure 3:**
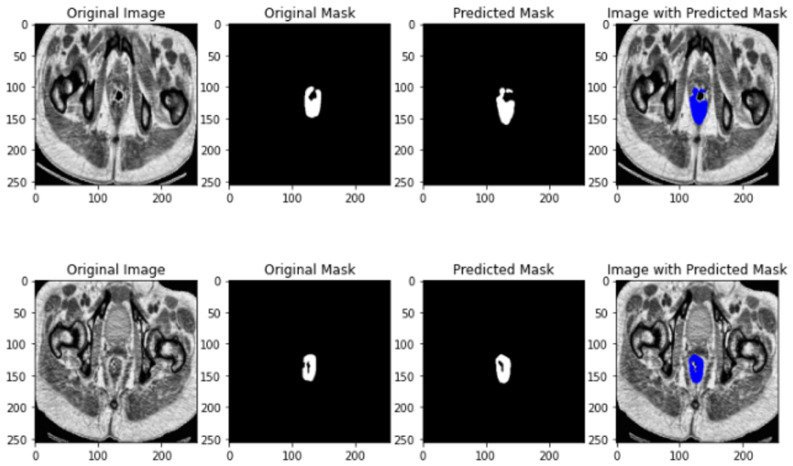
U-NET segmentation results.

The results of U-NET segmentation is depicted in Figure 3.

The feature map from the encoder is then passed through the decoder path, which uses up-convolutions with ReLU activations. Each block in the decoder concatenates the feature map from the encoder with the feature map in the decoder, resulting in increased spatial dimensions and reduced channels. The final output is a probability map that indicates the mask of the initial image, based on the generated feature maps.

##### U-NET++ [40]

Segmenting cancer lesions or any image in the medical domain in general demands a higher level of accuracy than what is desired in natural images. Marginal errors in segmentation can be critical to patients as they would heavily influence the doctor’s decision, especially if segmentation marks patterns around a cancerous part as invasive when they are not, which results in a lower credibility of the model from a clinical perspective. Therefore, the need to recover fine details in the medical domain proves to be crucial.

To address the need for more accurate representations, U-Net++ was implemented. In addition to having the same shape as the U-Net architecture, U-Net++ introduces nested and dense skip connections between encoder and decoder layers to effectively detect fine details that result from the encoder feature maps prior to fusion with the corresponding layer of the decoder. And this is the main difference with the U-Net architecture, which uses plain skip connections to fast-forward feature maps from encoder to decoder.

**Figure 4:**
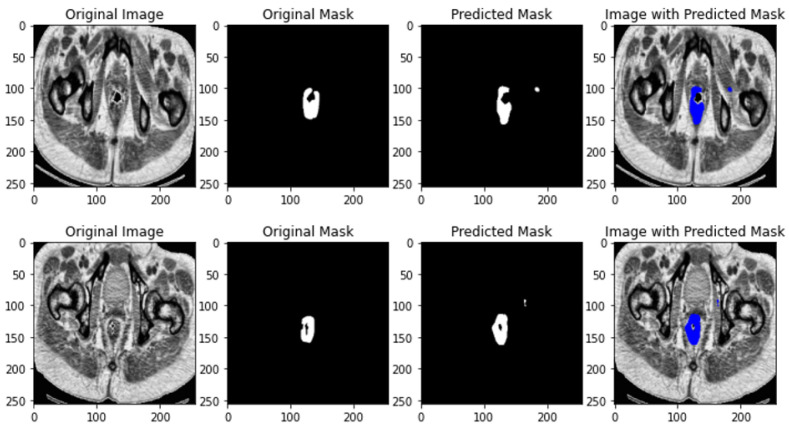
U-NET++ segmentation results.

The results of U-NET++ segmentation is depicted in Figure 4.

##### ResUNET [41]

As its name hints, ResUNET stands for residual U-NET. It was designed to obtain a high performance with fewer parameters compared to the existing architectures. It was an improvement over the U-NET series as it leverages the concept of deep residual networks in the context of U-NET. The use of residual blocks makes it possible to build a deeper network without worrying about the problem of vanishing or exploding gradients.

**Figure 5:**
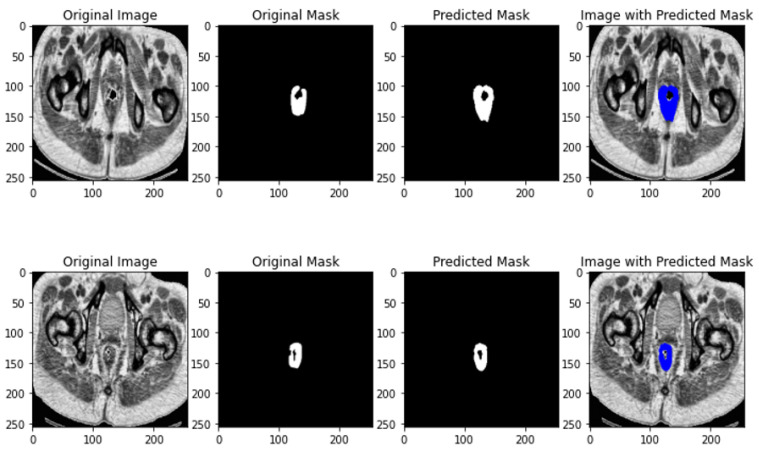
ResNET segmentation results.

The results of ResUNET segmentation is depicted in Figure 5.

##### ResUNET++ [42]

This architecture addresses the problem of the small number of images, which exists specifically the medical domain due to patient’s privacy. The ResUNET++ architecture takes advantage of not only residual blocks, but also squeeze and excitation blocks which increases the sensitivity to the relevant features and suppress all the unnecessary features, Spatial Pyramidal Pooling which will act as a bridge between the encode and decoder architecture to capture multi-scale information, and attention blocks that determine which parts of the network requires more attention. This DL architecture results in a significant improvement over the existing ResUNET architecture.

**Figure 6:**
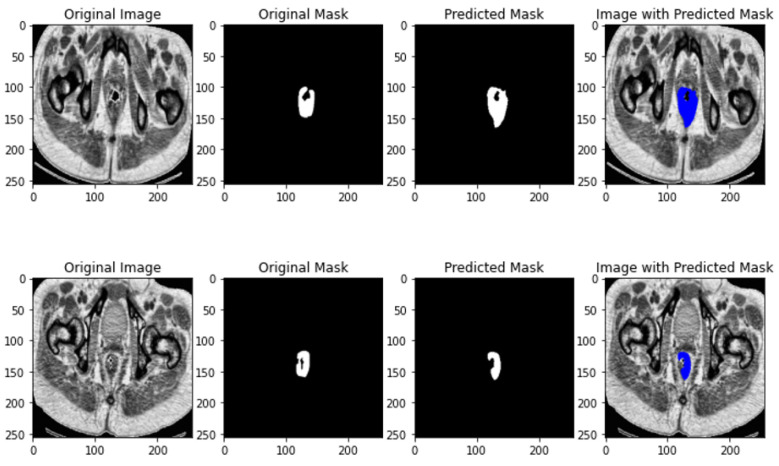
ResNET++ segmentation results.

**Figure 7:**
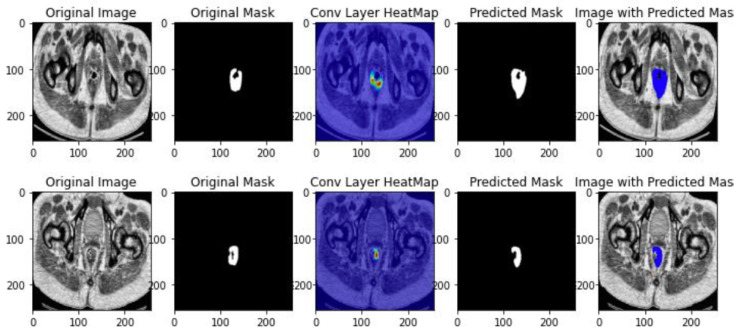
ResUNET++ class activation map.

The results of ResUNET++ segmentation is depicted in Figure 6 and Figure 7.

#### 4.3.2. Segmentation Quality and Validation

Table 8 presents the results of a segmentation experiment using four different models: UNET, UNET++, ResUNET, and ResUNET++. The evaluation was conducted using two metrics, namely DICE and IOU.

- **DICE Coefficient (Sørensen-Dice Index)**

The DICE coefficient measures the overlap between two sets and is commonly used in image segmentation tasks. The formula is

$$\mathrm{DICE}=\frac{2\times |A\cap B|}{\left|A|+|B\right|}$$

where

- *A* is the set of predicted labels (e.g., the predicted segmentation);
- *B* is the set of ground truth labels (e.g., the actual segmentation);
- |A∩B| is the intersection of the predicted and ground truth labels (i.e., the common area);
- |A| and |B| are the cardinalities (sizes) of sets *A* and *B*, respectively.

The DICE coefficient ranges between 0 and 1:

- A DICE coefficient of 1 indicates perfect overlap;
- A DICE coefficient of 0 indicates no overlap.

- **Intersection over Union (IOU)**

The IOU, also known as the Jaccard Index, is another metric for evaluating the overlap between two sets. The formula is

$$\mathrm{IoU}=\frac{\left|A\cap B\right|}{\left|A\cup B\right|}$$

where

- *A* is the predicted segmentation;
- *B* is the ground truth segmentation;
- |A∩B| is the intersection of sets *A* and *B*;
- |A∪B| is the union of sets *A* and *B*, which can be calculated as |A|+|B|−|A∩B|.

Like DICE, IoU also ranges between 0 and 1:

- An IoU of 1 means perfect overlap;
- An IoU of 0 means no overlap.

The results show that the ResUNET++ model achieved the highest performance with a DICE score of 76% and an IOU score of 61%. UNET and ResUNET also produced relatively high scores, with DICE scores of 74% and 72%, respectively, and IOU scores of 58% and 56%, respectively. UNET++ had the lowest scores with a DICE score of 67% and an IOU score of 51%.

These results suggest that ResUNET++ is the most effective model for segmentation in this experiment, as it achieved the highest scores in both metrics. It is also worth noting that the traditional UNET and ResUNET models also achieved relatively high performance, indicating that they are also viable options for segmentation tasks.

### 4.4. Multimodal Data Integration

Multimodal data integration is a critical process in healthcare analytics [43], involving the systematic combination of data from various sources or modalities. This integration is particularly significant in the context of CRC as it encompasses a broad spectrum of data types including clinical metrics, imaging scans, and patient-reported outcomes. Each of these modalities offers unique insights into the patient’s condition; when combined, they provide a comprehensive, multi-dimensional view of patient health that is greater than the sum of its parts.

The integration of these diverse data types serves several crucial functions. By correlating features across modalities, clinicians can enhance diagnosis and prognosis, identify patterns and markers that may be less apparent when considering each data type in isolation, and tailor treatments to individual patients based on a more complete understanding of their specific disease characteristics and predicted recovery trajectories. Additionally, for CRC patients, the period following surgery is fraught with uncertainties concerning recovery and QoL. Multimodal integration allows for more accurate predictions of these outcomes, aiding in better post-operative care and monitoring.

Thus, multimodal data integration not only enriches the data landscape but also enhances the actionable insights that can be derived from it, ultimately contributing to improved patient care and treatment outcomes in CRC.

Building on these principles, our study introduces a novel multimodal machine learning framework that strategically merges PCA-reduced image features with clinical data. By implementing principal component analysis, we effectively reduce the dimensionality of complex imaging data, retaining only the most critical features. These are then synergistically integrated with detailed clinical information, forming a unified dataset that feeds into a deep learning model. This approach not only capitalizes on the complementary strengths of each data modality but also addresses the challenges of data volume and computational efficiency, setting the stage for more accurate and robust predictive outcomes.

### 4.5. Results and Discussion

The results in Table 9 demonstrate the potential benefits of multimodal learning. For the GQOL12 target variable, there was no improvement in test accuracy when using a multimodal approach with joint fusion compared to a monomodal approach. However, for the Wexner Score 3, LARS Score 9, and AUR Score 9 target variables, the joint fusion approach outperformed the monomodal approach in terms of test accuracy.

This indicates that combining information from multiple modalities can lead to improved performance. For instance, the joint fusion approach achieved a significant improvement in the test accuracy of the AUR Score 9 target variable, going from 88% to 96%. This is particularly important as accurately predicting the AUR score can help clinicians make informed decisions about patient care.

Therefore, the results suggest that multimodal learning can provide valuable insights by leveraging complementary information from multiple sources. Those improvements can have a significant impact in real-world applications.

#### 4.5.1. Implications

The improvements in accuracy across various QoL indicators can be attributed to the synergistic effect of combining clinical data with CT image features in our multimodal approach. This integration allows our model to capture a more comprehensive picture of each patient’s condition, incorporating both observable clinical factors and underlying anatomical information.

By fusing these diverse data sources, our model can detect complex interactions between clinical variables and structural characteristics visible in CT scans. For instance, the relationship between tumor location, surrounding tissue composition, and post-operative functional outcomes may be more accurately captured, leading to improved predictions across multiple QoL domains. This approach also helps mitigate issues of data sparsity often encountered with clinical data alone, as imaging data provide a consistent set of features across all patients, potentially filling in information gaps.

The predictive model offers significant potential for improving post-operative management and long-term follow-up care in CRC patients. By providing more accurate forecasts of various QoL indicators, healthcare providers can tailor their care strategies to each patient’s specific needs and risks.

In the immediate post-operative period, the model’s predictions can guide the development of personalized rehabilitation programs. Patients predicted to have a higher risk of specific dysfunctions might be enrolled in more intensive therapy programs earlier in their recovery. For long-term follow-up care, the model enables a more proactive and personalized approach. By identifying patients at higher risk of experiencing poor QoL outcomes in specific domains, healthcare teams can implement targeted surveillance and intervention strategies.

#### 4.5.2. Limitations

Our study has several limitations that should be considered when interpreting the results. While our study included 100 patients, which is a relatively small sample size for machine learning applications, it is important to note that this represents a trade-off between sample size and data depth. The longitudinal nature of our study, with a one-year follow-up period and multiple assessment points per patient, provides rich, time-series data that capture the dynamic nature of QoL changes. This depth of data partially mitigates the limitations of a smaller sample size but may limit the generalizability of our findings to broader patient populations.

All patients were recruited from a single institution, which may introduce bias and limit the generalizability of our results to other healthcare settings or geographical regions. Multi-center studies would be valuable to validate our findings across diverse patient populations and healthcare systems. The retrospective nature of our study may introduce potential biases, particularly in terms of data completeness and quality. While we made every effort to ensure data integrity, prospective studies would be beneficial to further validate our multimodal approach.

Despite these limitations, our study provides valuable insights into the potential of multimodal machine learning for predicting QoL in CRC patients. Future research should aim to address these limitations through larger, multi-center prospective studies, incorporation of diverse imaging modalities, and continued refinement of model interpretability.

## 5. Conclusions

In the context of colorectal cancer management, the paradigm has increasingly shifted from a sole emphasis on survivability to a more holistic consideration of post-treatment QoL. Tumor resection, while a common therapeutic intervention, frequently does not yield significant improvements in patients’ QoL. This limitation has driven the research community to employ QoL questionnaires to capture patient outcomes over various time intervals. However, the absence of predictive models has constrained these assessments to largely descriptive analyses, which provide a generalized understanding of QoL trajectories without addressing specific health-related dimensions.

Furthermore, the existing literature predominantly focuses on descriptive statistics pertaining to global QoL measures, thereby neglecting the nuanced dimensions of health-related QoL, such as urinary, sexual, and functional outcomes. To address these gaps, our research introduces a novel system that utilizes empirical data to estimate multiple dimensions of health-related QoL prior to resection surgery. Notably, our approach includes the evaluation of urinary, sexual, and functional domains, offering a more granular analysis of anticipated patient outcomes. Additionally, we developed a segmentation model using image data from CT scans to accurately delineate affected anatomical regions, thereby enhancing the precision of our predictions.

This study makes a significant contribution to the existing body of knowledge by providing a more comprehensive and multidimensional assessment of health-related QoL in colorectal cancer patients. The integration of real-world data with advanced image-based segmentation techniques substantially increases the accuracy and robustness of our predictive system. The findings have critical implications for clinical practice, enabling healthcare providers to tailor treatment strategies more effectively to individual patient profiles. Ultimately, our work has the potential to significantly enhance the QoL of patients with colorectal cancer, thereby improving their overall well-being and reducing the burden of disease.

Future research could focus on expanding datasets to include more diverse patient populations across multiple institutions, improving model generalizability. Additionally, incorporating advanced deep learning architectures such as attention mechanisms or transformer-based models could enhance prediction accuracy by focusing on the most relevant features in clinical, imaging, and textual data [44].

Explainability [45] is another key area for improvement. Developing more interpretable models would help clinicians better understand the predictions and foster trust in AI-assisted decision-making. Lastly, future work could explore personalized intervention strategies based on model predictions, enabling early identification of at-risk patients and tailoring post-surgical care to improve outcomes.

## Figures and Tables

**Table 1:** Related works.

Article	Country	Objective	Data Types	Data Size	Pre-Processing	Feature Selection	Models	Main Metrics	Best Performance	Feature Importance
[20]	Netherlands	Descriptive Study Changes in QoL after tumor resection	Clinical	56	X	X	X	X	X	X
[21]	Scotland	Predictive Study Best Predictive Factors associated with QoL	Clinical	496	X	X	Linear Regression	R-Squared	0.574	Metastatic Gender Social functioning Fatigue Dyspnea Depression
[22]	Taiwan	Predictive Study postoperative recurrence risk among stage IV colorectal cancer patients	Clinical Demographical	999	Missing Data: Multiple Interpolation	X	Logistic Regression Decision Tree Gradient Boosting lightGBM	Main: AUC Secondary: Accuracy, F1-Score	LightGBM: AUC: 0.767 F1-Score: 0.974 Accuracy: 0.825	Chemotherapy Age Operation time Carcinoembryonic antigen radiotherapy
[23]	Lithuania	Descriptive Study long-term results of the QoL after surgical treatment of CRC	Clinical Demographical	88	X	X	Linear Regression	X	X	Age Tumor location
[24]	Brazil USA Japan Iran Spain Turkey Taiwan China	Systematic Review Short-Term Mortality Prediction for QoL Improvement	Clinical Demographical	173 to 26,946	Numerical: Normalization, Discretization, Standardization Categorical: One-hot encoding Missing Data: constant value, complete case only, Probalistic imputation, missForest	Zero Variance Correlation LASSO Recursive FS Forward FS	Decision Tree Neural Networks Naïve Bayes Gradient Boosting Linear Regressions Random Forest Support Vector Machine	Main: AUROC Secondary: Accuracy	Neural Networks: AUROC: 0.92 Accuracy: X	X
[25]	South Korea	Predictive Study HRQOL features in a 5-year lung cancer survival prediction	Clinical Sociodemographic	809	Data Balancing: Oversampling Missing Data Imputation	Forward Feature Selection	Decision Tree Bagging Random Forest AdaBoost Logistic Regression	Main: AUC Secondary: Accuracy	Bagging AUC: 0.981 Accuracy: 0.93	Cancer Stage Body Mass Index Depression Age
[26]	Sweden	Predictive Study prediction of QoL scores based on clinical data	Clinical	18,988 and 2466	Missing Data: Mean, Iterative MVI	X	Random Forest Support Vector Machine Naïve Bayes K Nearest Neighbor Decision Tree Neural Networks	F1-Score	Decision Tree and Random Forest F1-Score: 0.515–9.532	X
[27]	Finland Israel Portugal Italy	Predictive Study performance to predict patients’ QoL during treatment process	Sociodemographic Clinical and psychosocial	608	Target column discretization	X	Random Forest	Main: AUROC Secondary: Recall, Precision, Accuracy	AUROC: 0.832 Recall: 0.727 Precision:0.727 Accuracy: 0.785	Mental Health Age Distress Level Body Mass Index
[28]	Korea	Predictive Study gastric cancer recurrence after surgery	Clinical Demographical	2012	Missing Data Imputation Numerical Data: Normalization	X	lightGBM Random Forest Gradient Boosting Decision Tree Logistic Regression	Main: AUC Secondary: Accuracy, Precision, Recall	Random Forest AUC: 0.795 Recall: 0.148 Precision: 0.48 Accuracy: 0.797	Body Mass Index Weight Operation Time Age Height Tumor Size Chemotherapy Tumor Location
[29]	Taiwan	Predictive Study Risk factors associated with second cancer in CRC patients	Clinical	4287	X	Not Mentioned	Random Forest MARS XGBoost Support Vector Machine Extreme Learning Machine	Main: AUC Secondary: Accuracy	XGBoost: AUC: 0.723 Accuracy: 0.695	Sex Age Primary site Tumor Size Stage Drinking Behavior
[30]	Australia	Predictive Study Reduced HRQoL after tumor resection	Clinical	262	Missing Data: KNN Imputation Data Imbalance: SMOTE	X	Support Vector Machine Random Forest	AUC	SVM and Random Forest AUC: 0.69 to 0.98	X
[31]	Netherlands Germany	Segmentation Study Localization and Segmentation of rectal cancer	Magnetic Resonance Imaging	140	Standardization of intensities Data Augmentation	X	Neural Networks	Dice Coefficient	0.7	X

**Table 2:** Clinical data dictionary.

Feature Column	Description
Age	Patient’s age
Gender	Patient’s gender
AHT	Patient has arterial hypertension or not
Diabetes	Patient has diabetes or not
Cardiopathy	Patient has a heart disease or not
Operated	Patient was previously operated or not
Tobacco	Patient smokes or not
Alcohol	Patient drinks alcohol or not
S Case	Patient had a similar case in his family or not
Dig Tumor	Patient has a digestive tumor or not
Extradig Tumor	Patient has a tumor out of the digestive system or not
Hematochezia	Patient has hematochezia or not
Rectal Syd	Patient has rectal syndromes or not
Occlusion	Patient has intestinal obstruction or not
Diarrhea	Patient has diarrhea or not
Constipation	Patient has constipation or not
Other	Patient has clinical signs other than the mentioned ones or not
TR	Patient had rectal examination or not
TRaspect	The aspect of the rectal examination: ulcerative budding, stenosing, sphincter impairment
Distance	Anal margin
TR:contraction	Patient has contraction or not
TR:circumference	Rectal circumference: ½, ¾, circumferential
Fixity	Whether the tumor is fixed on an anterior or posterior organ
rectoig/colo	Whether the examination made is a recto sigmoidoscopy or colonoscopy
endo:pos	Tumor position from endoscopy examination
Stenosis	Stenosing tumor or not
Type his	The histological type corresponds to the normal cell from which derives the tumor
pos(TDM/TAP)	Tumor position from CT Scan
Extent	Tumor size estimation from CT Scan
envah	Whether the tumor is invasive or not
Resectability	Whether it is possible to resect the tumor or not
ADPs	Whether there is an adenopathy or not
META	Whether there is metastasis or not
CMT	Whether the patient has undergone chemotherapy or not
CCR	Whether the patient has concurrent chemo-radiotherapy or not
Protocol	The CCR protocol: short, intermediate, long
Delay	Delay between Post-CCR and surgery
Surgery Type	Surgery type: Colectomy, anterior rectal resection, abdominoperineal amputation with definitive or protective colostomy
Path	Laser surgery: Laparotomy, laparoscopy, laparo conversion
Anastomosis type	A surgical procedure in which the colon is attached to another organ after resection of the rectum.
Reservoir	Whether the patient has a reservoir or not (is infected by pathogens or not)
Ileostomy/colostomy	Whether the patient has an opening to divert toward the tummy or not.
Temporary colostomy	Whether the patient has temp colostomy or not
Definitive colostomy	Whether the patient has definitive colostomy or not
CMTadjuvant	Whether the patient has additional chemotherapy treatment to lower recurrence risk or not
ANAPATH	Whether there was an anatomical pathology or not
yTNM	The cancer classification
Complications	Whether the patient had postoperative complications or not
Fistula	Whether the patient has fistula or not

**Table 3:** Digestive troubles.

Target	Model	F1-Score	Acc	Selected Features
LARS score (1 month)	Random Forest	0.57	77%	Gender, Delay CMTadjuvante
LARS score (3 months)	LightGBM	0.51	73%	S Case, Extradig Tumor Constipation, pos(TDM/TAP) envah, Surgery Type Anastomosis type, Stade
LARS score (6 months)	Logistic Regression	0.34	63%	syd r, endo:pos ADPs, Surgery Type
LARS score (9 months)	Decision Tree	0.35	70%	TR:contracture, endo:pos pos(TDM/TAP), envah
Wexner Score (1 month)	K-Nearest Neighbors	0	67%	Age, Other endo:pos; Anastomosis type
Wexner Score (3 months)	K-Nearest Neighbors	0.40	67%	Operated, distance Delay, Anastomosis type
Wexner Score (6 months)	Decision Tree	0.30	77%	Occlusion, endo:pos Delay
Wexner Score (9 months)	Gradient Boosting	0.34	77%	TR:contracture, pos(TDM/TAP) Colostomy

**Table 4:** Urinary troubles.

Target	Model	F1-Score	Acc	Selected Features
UI Score (3 months)	XGB	0.21	70%	approach
UI Score (9 months)	XGB	0.33	77%	Gender, rectoig/colo CCR
AUR Score (3 months)	Random Forest	0.85	97%	Distance, Surgery Type approach
AUR Score (6 months)	Decision Tree	0.79	97%	syd r, Other endo:pos, Surgery Type approach
AUR Score (9 months)	Decision Tree	0.79	97%	pos(TDM/TAP), Protocol Anastomosis type, CMTadjuvante
AUR Score (12 months)	Gradient Boosting	0.91	93%	Extradig Tumor, approach colotomie IGD

**Table 5:** Sexual troubles—1.

Target	Model	F1-Score	Acc	Selected Features
Dyspareunia (3 months)	Bagging	0.18	63%	Constipation Fistula
Dyspareunia (6 months)	Decision Tree	0.31	77%	Gender, HTA Diabetes, Cardiopathy Operated, Tobacco Alcohol, S Case Extradig Tumor, rectorragies Occlusion
Dyspareunia (9 months)	Random Forest	0.70	90%	Gender, Alcohol Extradig Tumor, rectorragies syd r, Occlusion Other, Distance
Dyspareunia (12 months)	XGB	0.88	97%	Age Gender

**Table 6:** Sexual troubles—2.

Target	Model	F1-Score	Acc	Selected Features
Ejaculation Troubles	SVC	0.74	87%	Gender, Distance TR: circumference
Erection troubles (3 months)	K-Nearest Neighbor	0.91	97%	Gender, Diabetes Tobacco, syd r Constipation, envah
Erection troubles (6 months)	Random Forest	0.93	97%	Cardiopathy, Occlusion Distance, TR:circumference envah, resecability CCR, reservoir CMTadjuvante, ANAPATH
Erection troubles (9 months)	Decision Tree	0.82	93%	Gender, S Case Dig Tumor, rectorragies Constipation, Other Distance
Erection troubles (12 months)	Decision Tree	0.88	93%	Gender, syd r Diarrhea, Other Distance, TR:circumference

**Table 7:** Results—general quality of life.

Variable	Model	F1-1 Score	Acc	Selected Features
GQOL (1 month)	AdaBoost	0.85	87%	Age, Gender Cardiopathy, Operated Extradig Tumor, Diarrhea Other, stenose Protocol, approach ileotsomie, Temp Colostomy
GQOL (3 months)	XGB	0.84	83%	Gender, Extradig Tumor distance, Delay Colostomy
GQOL (6 months)	Gradient Boosting	0.8	80%	Extradig Tumor, pos(TDM/TAP) Anastomosis type, ileotsomie CMTadjuvante
GQOL (9 months)	XGB	0.87	87%	syd r, Occlusion Constipation, TRaspect rectoig/colo, endo:pos CCR, approach reservoir, Temp Colostomy Colostomy, Stade
GQOL (12 months)	Gradient Boosting	0.96	93%	HTA, Cardiopathy Operated, Tobacco Extradig Tumor, syd r Occlusion, Other endo:pos, type his envah, Protocol Delay, suite Fistula, Stade

**Table 8:** Segmentation results.

Model	DICE	IOU
UNET	74%	58%
UNET++	67%	51%
ResUNET	72%	56%
ResUNET++	76%	61%

**Table 9:** Multimodal vs. monomodal results.

Target Variable	Approach	Fusion Type	Test Accuracy
GQOL12	Mono-Modal	No Fusion	64%
	Multi-Modal	Joint Fusion	64%
Wexner Score 3	Mono-Modal	No Fusion	24%
	Multi-Modal	Joint Fusion	48%
LARS Score 9	Mono-Modal	No Fusion	60%
	Multi-Modal	Joint Fusion	60%
AUR Score 9	Mono-Modal	No Fusion	88%
	Multi-Modal	Joint Fusion	96%

## Data Availability

The data that support the findings of this study are available from the corresponding authors, upon reasonable request.

## References

[1] Sung H., Ferlay J., Siegel R.L., Laversanne M., Soerjomataram I., Jemal A., Bray F. (2021). Global cancer statistics 2020: GLOBOCAN estimates of incidence and mortality worldwide for 36 cancers in 185 countries. CA A Cancer J. Clin..

[2] Jansen L., Koch L., Brenner H., Arndt V. (2010). Quality of life among long-term (⩾5 years) colorectal cancer survivors–systematic review. Eur. J. Cancer.

[3] Saraswat D., Bhattacharya P., Verma A., Prasad V.K., Tanwar S., Sharma G., Bokoro P.N., Sharma R. (2022). Explainable AI for healthcare 5.0: Opportunities and challenges. IEEE Access.

[4] Chtouki K., Rhanoui M., Mikram M., Yousfi S., Amazian K. Supervised Machine Learning for Breast Cancer Risk Factors Analysis and Survival Prediction. Proceedings of the International Conference On Big Data and Internet of Things.

[5] Mikram M., Moujahdi C., Rhanoui M., Meddad M., Khallout A. Hybrid deep learning models for diabetic retinopathy classification. Proceedings of the International Conference On Big Data and Internet of Things.

[6] Ronneberger O., Fischer P., Brox T. U-net: Convolutional networks for biomedical image segmentation. Proceedings of the International Conference on Medical Image Computing and Computer-Assisted Intervention.

[7] Fu Y., Lei Y., Wang T., Curran W.J., Liu T., Yang X. (2021). A review of deep learning based methods for medical image multi-organ segmentation. Phys. Medica.

[8] Choi M.S., Choi B.S., Chung S.Y., Kim N., Chun J., Kim Y.B., Chang J.S., Kim J.S. (2020). Clinical evaluation of atlas-and deep learning-based automatic segmentation of multiple organs and clinical target volumes for breast cancer. Radiother. Oncol..

[9] Xie Y., Xing F., Shi X., Kong X., Su H., Yang L. (2018). Efficient and robust cell detection: A structured regression approach. Med Image Anal..

[10] Mahmood F., Borders D., Chen R.J., McKay G.N., Salimian K.J., Baras A., Durr N.J. (2019). Deep adversarial training for multi-organ nuclei segmentation in histopathology images. IEEE Trans. Med Imaging.

[11] Takamatsu M., Yamamoto N., Kawachi H., Chino A., Saito S., Ueno M., Ishikawa Y., Takazawa Y., Takeuchi K. (2019). Prediction of early colorectal cancer metastasis by machine learning using digital slide images. Comput. Methods Programs Biomed..

[12] Bychkov D., Linder N., Turkki R., Nordling S., Kovanen P.E., Verrill C., Walliander M., Lundin M., Haglund C., Lundin J. (2018). Deep learning based tissue analysis predicts outcome in colorectal cancer. Sci. Rep..

[13] Baltrušaitis T., Ahuja C., Morency L.P. (2018). Multimodal machine learning: A survey and taxonomy. IEEE Trans. Pattern Anal. Mach. Intell..

[14] Lee S.I., Yoo S.J. (2020). Multimodal deep learning for finance: Integrating and forecasting international stock markets. J. Supercomput..

[15] Bousnina N., Ghouzali S., Mikram M., Abdul W. DTCWT-DCT watermarking method for multimodal biometric authentication. Proceedings of the 2nd International Conference on Networking, Information Systems & Security.

[16] Acosta J.N., Falcone G.J., Rajpurkar P., Topol E.J. (2022). Multimodal biomedical AI. Nat. Med..

[17] Borgaonkar M., Irvine E. (2000). Quality of life measurement in gastrointestinal and liver disorders. Gut.

[18] Lewandowska A., Rudzki G., Lewandowski T., Próchnicki M., Rudzki S., Laskowska B., Brudniak J. (2020). Quality of life of cancer patients treated with chemotherapy. Int. J. Environ. Res. Public Health.

[19] Velikova G., Booth L., Smith A.B., Brown P.M., Lynch P., Brown J.M., Selby P.J. (2004). Measuring quality of life in routine oncology practice improves communication and patient well-being: A randomized controlled trial. J. Clin. Oncol..

[20] Breukink S., Van der Zaag-Loonen H., Bouma E., Pierie J., Hoff C., Wiggers T., Meijerink W. (2007). Prospective evaluation of quality of life and sexual functioning after laparoscopic total mesorectal excision. Dis. Colon Rectum.

[21] Gray N.M., Hall S.J., Browne S., Macleod U., Mitchell E., Lee A.J., Johnston M., Wyke S., Samuel L., Weller D. (2011). Modifiable and fixed factors predicting quality of life in people with colorectal cancer. Br. J. Cancer.

[22] Xu Y., Ju L., Tong J., Zhou C.M., Yang J.J. (2020). Machine learning algorithms for predicting the recurrence of stage IV colorectal cancer after tumor resection. Sci. Rep..

[23] Valeikaite-Tauginiene G., Kraujelyte A., Poskus E., Jotautas V., Saladzinskas Z., Tamelis A., Lizdenis P., Dulskas A., Samalavicius N.E., Strupas K. (2022). Predictors of quality of life six years after curative colorectal cancer surgery: Results of the prospective multicenter study. Medicina.

[24] Lu S.C., Xu C., Nguyen C.H., Geng Y., Pfob A., Sidey-Gibbons C. (2022). Machine Learning–Based Short-Term Mortality Prediction Models for Patients With Cancer Using Electronic Health Record Data: Systematic Review and Critical Appraisal. JMIR Med. Inform..

[25] Sim J.A., Kim Y., Kim J.H., Lee J.M., Kim M.S., Shim Y.M., Zo J.I., Yun Y.H. (2020). The major effects of health-related quality of life on 5-year survival prediction among lung cancer survivors: Applications of machine learning. Sci. Rep..

[26] Savić M., Kurbalija V., Ilić M., Ivanović M., Jakovetić D., Valachis A., Autexier S., Rust J., Kosmidis T. Analysis of Machine Learning Models Predicting Quality of Life for Cancer Patients. Proceedings of the 13th International Conference on Management of Digital EcoSystems.

[27] Nuutinen M., Korhonen S., Hiltunen A.M., Haavisto I., Poikonen-Saksela P., Mattson J., Kondylakis H., Mazzocco K., Pat-Horenczyk R., Sousa B. Impact of machine learning assistance on the quality of life prediction for breast cancer patients. Proceedings of the 15th International Joint Conference on Biomedical Engineering Systems and Technologies.

[28] Zhou C., Hu J., Wang Y., Ji M.H., Tong J., Yang J.J., Xia H. (2021). A machine learning-based predictor for the identification of the recurrence of patients with gastric cancer after operation. Sci. Rep..

[29] Ting W.C., Chang H.R., Chang C.C., Lu C.J. (2020). Developing a novel machine learning-based classification scheme for predicting SPCs in colorectal cancer survivors. Appl. Sci..

[30] Karri R., Chen Y.P.P., Drummond K.J. (2022). Using machine learning to predict health-related quality of life outcomes in patients with low grade glioma, meningioma, and acoustic neuroma. PLoS ONE.

[31] Trebeschi S., van Griethuysen J.J., Lambregts D.M., Lahaye M.J., Parmar C., Bakers F.C., Peters N.H., Beets-Tan R.G., Aerts H.J. (2017). Deep learning for fully-automated localization and segmentation of rectal cancer on multiparametric MR. Sci. Rep..

[32] Peterson L.E. (2009). K-nearest neighbor. Scholarpedia.

[33] Whitley D. (1994). A genetic algorithm tutorial. Stat. Comput..

[34] Yang X.S. Flower pollination algorithm for global optimization. Proceedings of the International Conference on Unconventional Computing and Natural Computation.

[35] Mirjalili S. (2016). SCA: A sine cosine algorithm for solving optimization problems. Knowl.-Based Syst..

[36] Mirjalili S., Gandomi A.H., Mirjalili S.Z., Saremi S., Faris H., Mirjalili S.M. (2017). Salp Swarm Algorithm: A bio-inspired optimizer for engineering design problems. Adv. Eng. Softw..

[37] Schapire R.E. (2013). Explaining adaboost. Empirical Inference: Festschrift in Honor of Vladimir N. Vapnik.

[38] Chen T. (2015). Xgboost: Extreme gradient boosting. R Package Version 0.4-2.

[39] Friedman J.H. (2001). Greedy function approximation: A gradient boosting machine. Ann. Stat..

[40] Zhou Z., Rahman Siddiquee M.M., Tajbakhsh N., Liang J. (2018). Unet++: A nested u-net architecture for medical image segmentation. Deep Learning in Medical Image Analysis and Multimodal Learning for Clinical Decision Support.

[41] Diakogiannis F.I., Waldner F., Caccetta P., Wu C. (2020). ResUNet-a: A deep learning framework for semantic segmentation of remotely sensed data. ISPRS J. Photogramm. Remote Sens..

[42] Jha D., Smedsrud P.H., Riegler M.A., Johansen D., De Lange T., Halvorsen P., Johansen H.D. Resunet++: An advanced architecture for medical image segmentation. Proceedings of the 2019 IEEE International Symposium on Multimedia (ISM).

[43] Bardak B., Tan M. (2021). Improving clinical outcome predictions using convolution over medical entities with multimodal learning. Artif. Intell. Med..

[44] Zekaoui N.E., Yousfi S., Mikram M., Rhanoui M. Enhancing Large Language Models’ Utility for Medical Question-Answering: A Patient Health Question Summarization Approach. Proceedings of the 2023 14th International Conference on Intelligent Systems: Theories and Applications (SITA).

[45] Tjoa E., Guan C. (2020). A survey on explainable artificial intelligence (xai): Toward medical xai. IEEE Trans. Neural Netw. Learn. Syst..

